# Cations Responsible for Varied CO_2_RR Product Selectivity at High Overpotentials over Cu_2_O Nanocubes

**DOI:** 10.1021/acsami.5c09803

**Published:** 2025-09-02

**Authors:** Saeede Tafazoli, Azra Şekercioğlu, Hamaneh Zarenezhad, Başak Ataş, Amin Mohammadpour, Timuçin Balkan, Sarp Kaya

**Affiliations:** † Materials Science and Engineering, 52979 Koç University, Istanbul 34450, Türkiye; ‡ 322088 Koç University Hydrogen Technologies Center (KUHyTech), Istanbul 34450, Türkiye; § Department of Chemistry, Koç University, Istanbul 34450, Türkiye

**Keywords:** CO_2_ reduction reaction, Cu_2_O nanocubes, cations, product selectivity, electrochemical spectroscopy, catalyst optimization

## Abstract

This study investigates the role of cations in tuning the structure and chemical state of Cu_2_O nanocubes during CO_2_ reduction reactions (CO_2_RR) and how these changes impact product selectivity. Different cations (Li^+^, K^+^, Cs^+^) in carbonate $\left({\mathrm{CO}}_{3}^{2-}\right)$ electrolytes have a significant effect on the dissolution and reprecipitation of Cu species, which alters the surface chemistry of electrodes. Li^+^ promotes the dissolution of Cu, leading to a metallic surface that favors CH_4_ production, while K^+^ and Cs^+^ stabilize the Cu oxide and hydroxide species on the surface, resulting in higher faradaic efficiency for C_2_H_4_. A combination of ex situ and in situ techniques, including X-ray absorption spectroscopy (XAS), X-ray photoelectron spectroscopy (XPS), and electrochemical surface-enhanced Raman spectroscopy (EC-SERS), demonstrates cation-induced changes in the Cu oxidation state and surface structure directly affecting CO_2_RR product selectivity.

## Graphical abstract

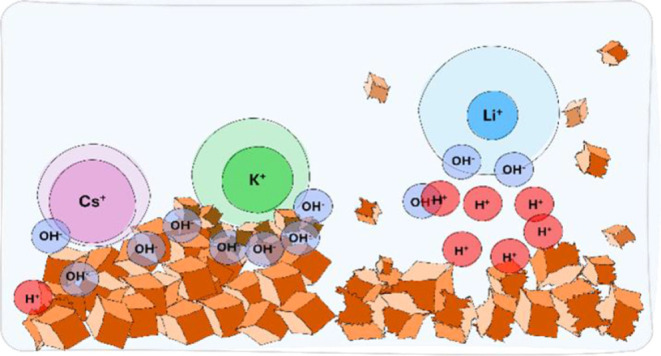

## Introduction

The chemical conversion of CO_2_ presents a promising approach to producing valuable chemicals and closing the carbon cycle using renewable energy sources. Due to increasing energy demands, CO_2_ reduction reactions (CO_2_RR) have gained significant attention over the past decade.^1–4^ Copper (Cu) stands out among active metals for CO_2_RR, due to its unique ability to convert CO_2_ into various hydrocarbons, including methane (CH_4_) and ethylene (C_2_H_4_), by leveraging optimal binding energies for adsorbed hydrogen (H_ad_) and carbon monoxide (CO_ad_).^5^

Meanwhile, the microstructure and chemical state of Cu play critical roles in determining the intermediates and product selectivity of CO_2_RR. Key structural parameters such as grain size, shape, roughness, active surface area, and crystal facets significantly influence product selectivity. For example, Cu (111) is active in producing C_2_H_4_ and CH_4_ at higher overpotentials, whereas Cu (100) primarily yields C_2_H_4_ at lower overpotentials.^6,7^ Furthermore, the oxidation state of Cu is crucial in defining binding energies and product pathways. For instance, a combination of Cu^+^ and Cu^0^ is essential for producing longer carbon chain products (C2+).^8^ However, the presence of Cu^2+^ in the form of Cu hydroxide (Cu­(OH)_2_) enhances faradaic efficiency (FE) for C_2_H_4_. Research by Xu group^9^ has shown that specific active sites can have distinct selectivity between C1 and C2+ products. High selectivity for CH_4_ has also been reported when Cu^2+^ is introduced into the electrolyte to compensate for the dissolved Cu.^10^ This selectivity is attributed to the restructuring of the surface in the presence of Cu ions in the solution.

The role of electrolytes in product selectivity is critical due to their influence on reactions occurring at the electrode/electrolyte interface.^11–13^ Understanding how the electrolyte affects the electrode surface in the presence of reactants and intermediates of CO_2_RR and hydrogen evolution reaction (HER), as the side reaction in aqueous electrolytes, is essential. During CO_2_RR, the negatively charged surface of Cu can rearrange the presence of cations near the electrode surface. Recent studies have investigated the impact of alkali metal cations on active metals for CO_2_RR such as silver (Ag),^14,15^ gold (Au),^16^ and Cu. Hori and coworkers^17^ previously found that product distribution depends on the nature of the cation, with a higher C2+/C1 product ratio observed in the presence of weakly hydrated cations such as Cs^+^ and K^+^ compared to Li^+^. This phenomenon has been attributed by Hori and coworkers to the lower local pH and higher proton concentration associated with Li^+^, promoting CO protonation and leading to the formation of CH_4_. Similarly, high CH_4_ selectivity has been reported in the presence of strongly hydrated cations, particularly Na^+^.^18,19^

Several mechanisms have been previously proposed to explain the effect of the electrolyte on CO_2_RR rates and product selectivity include: (a) local buffering by hydrated cations, (b) cation concentration in the double-layer region, and (c) potential redistribution affecting adsorbed intermediates.^20^ However, these explanations are not fully consistent with CO_2_RR behavior under various conditions, as we discuss in more detail below. Previous studies on Ag and Au have proposed that weakly hydrated cations can tune the local pH in favor of higher activity for CO_2_RR to CO and suppression of HER, although this finding contradicts enhanced HER on Cu electrodes in the presence of Cs^+ 21^. It has also been proposed that the interfacial electric field and cation distance from the electrode surface significantly influence the potential distribution and stabilization of intermediates, which can lead to specific product distribution. Xu and his coworkers highlighted that cation-induced electric and nonelectric field factors might affect the composition and structure of the electrolyte and catalyst interface resulting in different adsorption sites for CO, though direct experimental evidence of this effect on the catalyst is lacking.^21^ Despite extensive research, the cation effects presented above and whether they can alter the surface structure and contribute to the specific product selectivity remain unknown. Thus, the interplay between cations, structural changes, and product selectivity is not yet fully understood.

This study presents ex situ and in situ experimental results demonstrating the significant impact of the cations on the structure and chemical state of Cu_2_O nanocubes (NCs). Product selectivity of CO_2_RR varied with the type of cation in the electrolyte at higher overpotentials. Li^+^ favored CH_4_ production, while Cs^+^ and K^+^ favored C_2_H_4_. Investigations on the electrode surface during and after CO_2_RR in electrolytes containing these cations revealed that metallic Cu was the primary phase at high overpotential in the presence of Li^+^. Employing rotating ring disk electrode (RRDE) techniques clarified that the formation of metallic Cu as the major phase in the presence of Li^+^ is caused by higher proton concentration due to the interfacial electric field and potential distribution imposed by Li^+^. Higher proton concentration led to Cu dissolution from the surface at high cathodic potentials, which subsequently redeposited as a metallic phase. However, Cu^+^ and Cu^2+^ (Cu­(OH)_2_) were the main phases in the presence of Cs^+^ and K^+^, respectively. These phases and their ratios were confirmed using electrochemical surface-enhanced Raman spectroscopy (EC-SERS) under potential (in situ) and postreaction surface analyses. Our findings underscore the critical role of cations in dictating the structure and chemical state of Cu oxide (Cu_2_O) NCs during CO_2_RR, thereby influencing product selectivity. This interplay provides a deeper understanding of CO_2_RR mechanisms over Cu oxide electrodes at higher cathodic potentials, paving the way for optimizing more stable electrocatalysts with desired product selectivity.

## Experimental Methods

### Electrode Preparation

Cu_2_O NCs were synthesized through a wet chemistry process. l-Ascorbic acid (C_6_H_8_O_6_) was used as a reductant to avoid the need for a surfactant and to prevent any unwanted effect of surfactant residues on the surface of the NCs.^22,23^ To synthesize 100 nm NCs, 4 mL of 0.2 M CuSO_4_ (≥99%, Sigma-Aldrich) was added to a beaker containing 366 mL H_2_O on a stirrer. To initiate the nucleation, 14 mL of 3 M NaOH (≥99.0%, ISOLAB) was added to the stirring solution. After 10 s, 16 mL of 0.25 M l-ascorbic acid (Sigma-Aldrich-reagent grade) was added. The solution was stirred for an additional 13 min. Then it was left undisturbed for 1 h to allow the NCs to precipitate at the bottom of the beaker. The NCs were then cleaned by washing five times, three with an ethanol–water mixture (1:1) and two with only ethanol using a centrifuge. The obtained NCs were dried and then mixed with 10:1 ethanol to 5 wt % Nafion and drop-casted onto a gas diffusion electrode (GDE) or a glassy carbon (GC) substrate, with identical loading of 1.1 mg/cm^2^. Then, the drop-casted electrode was dried at 60 °C on a heater.

### Electrode Characterization

Morphology of the surface of the electrodes before and after CO_2_RR, was examined through field emission scanning electron microscopy (FESEM, Zeiss Ultra Plus – 20 kV). To investigate the chemical state of the electrodes before and after CO_2_RR at the near-surface region, X-ray photoelectron spectroscopy (XPS) and X-ray absorption spectroscopy (XAS) at Cu L edge and O K edge measurements were performed. XPS measurements (Thermo K-alpha) were performed using Al K_α_ (hν = 1486.7 eV) X-ray source. Electrodes were rinsed with deionized (DI) water and dried with N_2_ to prevent any contamination affecting the results. XAS measurements were performed at the HESEB Beamline SESAME (Synchrotron-Light for Experimental Science and Applications in the Middle East, Allan, Jordan). All spectra was normalized by incident photon flux.

### Electrochemical Measurements

Electrochemical CO_2_RR measurements were conducted in a customized H-type electrochemical cell. Potentials were applied using an SP-200 BioLogic potentiostat (chronoamperometry method). A three-electrode setup with Cu_2_O NCs coated GDE electrodes as working electrodes (WE), an Ag/AgCl (saturated KCl) as a reference electrode (RE), and a graphite rod as a counter electrode (CE) was used. The electrolytes (0.1 M M_2_CO_3_, M = Cs^+^, K^+^, Li^+^) were prepared and saturated with CO_2_ before and during the CO_2_RR.

All potentials were measured versus a Ag/AgCl reference electrode and converted to reversible hydrogen electrode (RHE) potential using Nernst equation:

$$E_{\mathrm{RHE}}{=E}_{\mathrm{Ag}/\mathrm{AgCl}}+\left(0.059\times \mathrm{pH}\right)+E_{\mathrm{Ag}/\mathrm{AgCl}}^{{}^{\circ}}$$

The thermodynamic standard reduction potential, E°_Ag/AgCl_, of Ag/AgCl electrode is measured 0.197 V. The uncompensated resistance of the electrolyte was measured through the electrochemical impedance spectroscopy (EIS) method at single-point impedance at 100 kHz automatically before chronoamperometry (CA) and 85% of the *iR*_u_ drop (i: current) was corrected.

Gas products of the CO_2_RR on Cu_2_O NCs were detected and quantified using a gas chromatograph (GC, Agilent 7820A) equipped with a thermal conductivity detector (TCD) and a flame ionization detector (FID). All GC measurements were conducted with continuous purging of CO_2_, and total head space volume (*V*_total_) was calculated using a mass flow controller as follows:

$$V_{\mathrm{total}}\;\left(\mathrm{mL}\right)=\mathrm{duration of the experiment}\;\left(\min\right)\times \mathrm{flow rate of gas}\;\left(\mathrm{mL}/\min\right)$$

For FE calculation, the number of moles of the product was derived by applying the ideal gas law under standard conditions:

$$n_{\mathrm{total}}=\frac{V_{\mathrm{total}}}{22400}$$

For each gas, the concentration was calculated using the standard gas mixture calibration:

$$C_{x}=\left(\frac{{\mathrm{Peak area of gas x obtained during CO}}_{2}\mathrm{RR}}{\mathrm{Peak area of gas x in the standard gas mixture}}\right)\times \left(\mathrm{Volumetric \% of gas x in the standard gas mixture}\right)$$

From total moles of gases in the headspace, number of moles of each gaseous component was calculated using the obtained concentration coefficient (C_x_):

$$n_{x\left(\mathrm{flow}\right)}=C_{x}{\times n}_{\mathrm{total}}$$

Finally, the FE of the gaseous product was calculated using the following formula:

$${\mathrm{FE}}_{x}=\frac{n_{x\left(\mathrm{flow}\right)}\times n\times F}{Q_{\mathrm{total}}}$$

In this equation, n represents the number of electrons in that reaction, F is the Faraday constant, and *Q*_total_ is the total charge passed through the system.

The rotating ring disk electrode (RRDE, Pine Research Instrumentation) technique was used to detect the dissolution of Cu during the CO_2_RR at varied conditions. RRDE tip contains a GC disk and a Pt ring with diameters of 5.0 mm, and outer and inner diameters of 7.5 mm and 6.5 mm, respectively. The disk and the ring are isolated from each other by a Teflon ring. Initially, both disk and tip were polished using a microcloth with 0.5 and then 0.05 μm alumina slurry applied. Then, the Pt ring was electropolished through cycling between 0 and 1.6 V at 500 mV/s for 100 cycles in 0.5 M H_2_SO_4_. The surface of the GC disk was covered by prepared Cu_2_O NCs ink drop-cast (5 μL) and subsequently was inserted using the touch-free accessories. For experiments performed at open circuit potential (OCP), the disk was not under potential and the ring was cycled between 0 to 1.6 V vs RHE. The potential was applied through a potentiostat (WaveDriver 200, Pine Research Instrumentation) and an MSR rotator was employed and rotate the tip (disk and ring) at 1600 rpm to provide centrifugal force. The concentration of the dissolved Cu during the CO_2_RR in H-cell was determined by the inductively coupled plasma- mass spectrometry method (ICP-MS, Agilent 7700x). ECSA calculation was performed following the procedure described in our previous study.^24^

EC-SERS was performed using a Renishaw INVIA Raman system with a long working distance (8 mm −50×) objective. Cu_2_O NCs provide a SERS active surface due to their partial or full reduction under the application of cathodic potential. Raman spectra were generated using a 632.8 nm laser. EC-SERS investigations were performed in a customized electrochemical cell.^24^ The system was calibrated at the beginning of the measurements using Si (100) wafer. No baseline subtraction was done on the Raman spectra. For the three-electrode setup, a Pt wire as the CE and an Ag/AgCl electrode as the RE were used. Potentials were applied to an SP-200 BioLogic potentiostat.

## Results and Discussion

First, the effect of different alkali metal cations on CO_2_RR selectivity using Cu_2_O NCs was investigated. Results clearly demonstrated an increased selectivity toward CH_4_ (27%) in the presence of Li^+^ and an enhanced formation of C_2_H_4_ (ca. 30–33%) in the presence of K^+^ and Cs^+^ (Figure 1) especially at high overpotential, consistent with previous studies conducted on metallic Cu^17^. This dependency on the nature of the cation is attributed to several factors: Hori and coworkers^17^ noted that differences in the outer Helmholtz plane (OHP), which are dependent on the size of the cation, lead to varied proton concentrations at the electrode–electrolyte interface. Cations of different sizes could also induce an electric field that can stabilize reaction intermediates critical to selective product formation. Moreover, the strength of the cations to hydrolysis could be crucial, where they act as a buffering agent and adjust local pH.

**Figure 1:**
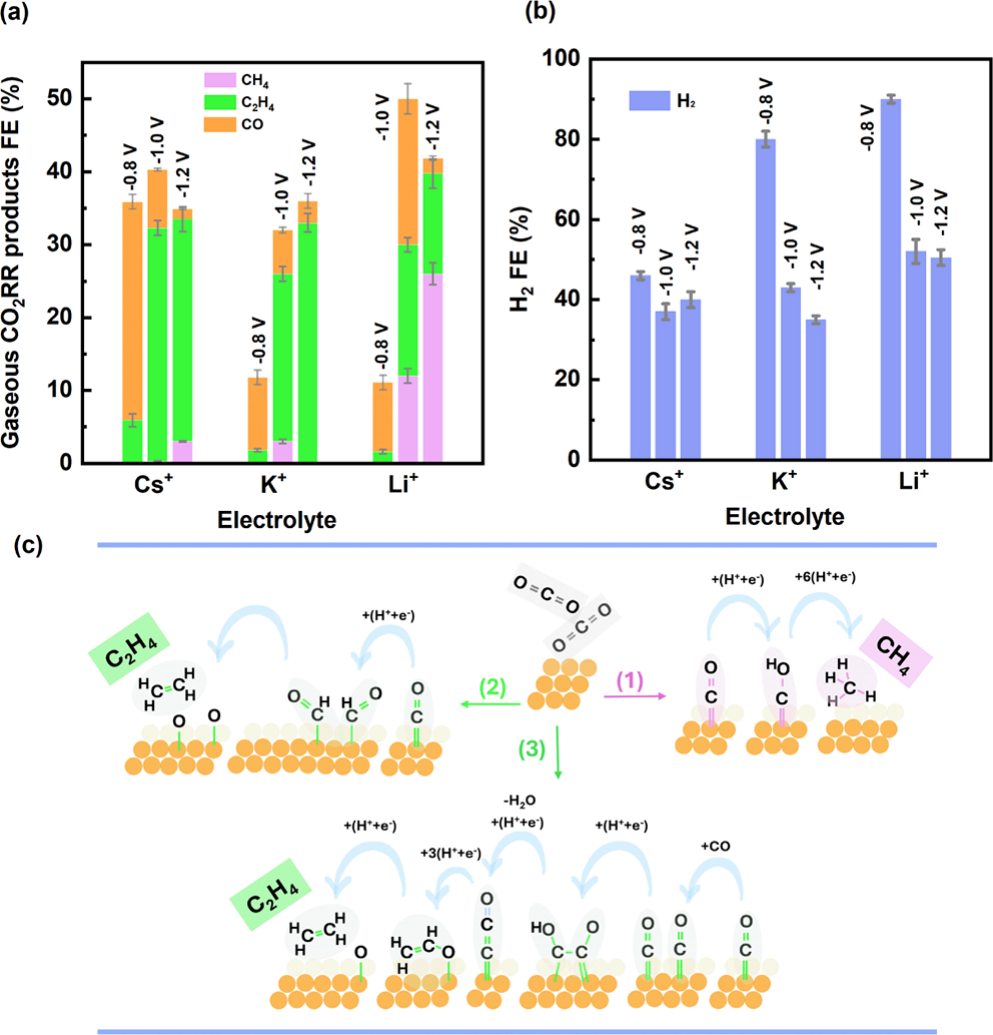
Gaseous products of (a) CO_2_RR and (b) HER on Cu_2_O NCs in CO_2_ purged 0.1 M Cs_2_CO_3_, K_2_CO_3_, and Li_2_CO_3_ electrolytes, and (c) pathways of CO_2_RR to CH_4_ and C_2_H_4_. Indicated potentials are reported vs. RHE.

The highest FE of CH_4_ and C_2_H_4_ was obtained at the highest applied potential, −1.2 V. At lower cathodic potentials of −0.8 and −1.0 V, selectivity did not show a considerable difference depending on the cations; H_2_ and CO are found to be the major products. Figure 1c presents a simplified schematic showing the main pathways at low and high cathodic potentials toward CH_4_ and C_2_H_4_. A more complete mechanism, including all intermediate steps, can be found in detailed CO_2_RR pathway reviews.^25,26^ From a mechanistic perspective, CH_4_ and C_2_H_4_ share a high-overpotential pathway through a common intermediate, CHO_ad_.^27^ This pathway occurs on Cu(111) and Cu(100) facets^7,25^ (Figure 1c, pathway 1 and 2). Another pathway predicted for C_2_H_4_ (Figure 1c, pathway 3) involves the dimerization when two CO_ad_ molecules form a C–C bond. This pathway occurs at lower overpotentials on Cu(100) and it is pH insensitive. The formation of C_2_H_4_ through the dimerization pathway depends on the availability of adsorption sites for CO.^7,28,29^

It has been proposed that a lower pH, where the chance of protonation of CO_ad_ as the rate-determining step (RDS) is higher, favors the formation of CH_4_ over C_2_H_4_. However, the first pathway, which CH_4_ and C_2_H_4_ share, is also believed to depend on local pH,^30^ suggesting that changes in local pH could affect both products.

Despite this, our results indicate that the formation of CH_4_ at high overpotentials contradicts the simple low-pH hypothesis. At high cathodic potentials, elevated CO_2_RR and HER rates increase current density and thus OH^–^ concentration,^31^ which would raise local pH, not lower it. Moreover, according to Kas and coworkers,^30^ at a higher local pH, when bicarbonate as a strong buffering agent is at a lower concentration, the formation of C_2_H_4_ is favored over CH_4_. These contradictions indicate that the selectivity of CH_4_ and C_2_H_4_ cannot be explained by the local pH-based mechanism proposed in previous studies alone and require further investigation. It is also worth noting that, as shown in Figure 1b, we observe that higher C_2_H_4_ selectivity is accompanied by lower H_2_ formation. This can be explained by the fact that OH^–^ generated at higher cathodic potentials accumulate on the Cu surface, lowering the CO–CO coupling barrier and favoring C2 product formation.^30,32^ At the same time, the presence of interfacial OH^–^ blocks proton access, suppressing the competing hydrogen evolution reaction.

Current densities recorded during CO_2_RR in three different electrolytes are compared in Figure S1. At −1.2 V, current densities for NCs in K^+^ and Li^+^ electrolytes are similar but lower than those in the Cs^+^. Since higher concentrations of OH^–^ are generated at higher current densities^17,31^ it is expected that the local pH would be similar for the electrodes in Li^+^ and K^+^ electrolytes, provided that the cations did not affect the buffering. However, this observation does not align with the observed selectivity at −1.2 V and contradicts the previously proposed effect of local pH on selectivity. Additionally, Singh^14^ and coworkers reported that the p*K*_a_ of cations near the electrode surface decreases in the order of Cs^+^ < K^+^ < Li^+^, implying that Li^+^ leads to a higher pH because it donates protons less readily to OH^–^. Consequently, CO_2_ could be depleted near the surface by reaction with the excess OH^–^. However, our data show that only C_2_H_4_ is strongly suppressed while CH_4_ remains favored in Li^+^, which does not agree with the local pH hypothesis either.

To elucidate the influence of cations on surface structure and to gain deeper insight into their role in selectivity, we examined the surface of the electrodes before and after CO_2_RR in the presence of three different cations.

FESEM images (Figure 2b–d) reveal that NCs undergo significant surface reconstruction and shrinkage after CO_2_RR at −1.2 V compared to the as-prepared electrode surface (Figure 2a). The most notable changes were observed in the presence of Li^+^. This effect is even more pronounced for larger nanocubes (400 nm) in the presence of Li^+^ (Figure S2), where an entirely new surface structure is formed on the NCs. This restructuring likely results from the dissolution and redeposition of Cu during CO_2_RR, influenced by CO_2_RR intermediates and the applied potential.^33–36^ Our FESEM investigations indicate that this restructuring can be cation-dependent.

**Figure 2:**
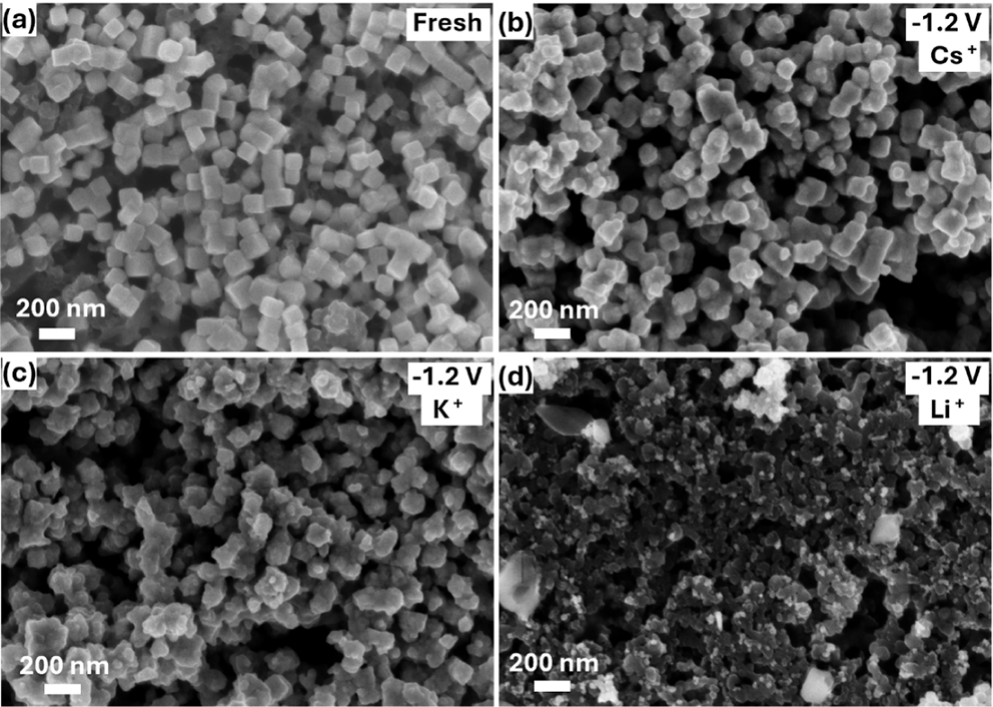
Morphological characterization of Cu_2_O nanocubes, (a) before and after CO_2_RR in CO_2_-purged 0.1 M of (b) Cs_2_CO_3_, (c) K_2_CO_3_, and (d) Li_2_CO_3_ at −1.2 V.

During CO_2_RR, the structure of the Cu surface could be influenced by reaction intermediates, specifically CO.^33,37–39^ FESEM images (Figure S3a–c) of electrodes that electrolyzed the CO_2_RR at −0.8 V in a Cs^+^ containing electrolyte vividly illustrate the effect of CO, which is the main product at this potential (Figure 1a). It could be argued that the bright clusters observed on the surface of this electrode are likely to be induced and/or stabilized by the CO. Incidentally, it has been shown that CO on the low-coordinated Cu adatoms gives rise to dynamic changes in the surface sctructure.^37,39^ The trend in surface restructuring at −1.2 V is opposite to that observed at −0.8 V. FESEM images taken after exposing NCs to open circuit potential (OCP) conditions (Figure S3d–f) indicate that the morphology of the NCs is not affected by the nature of the cation at this potential.^40^ However, the observed surface morphology cannot be attributed solely to CO; H_2_ evolution, local pH variations, as well as the dissolution and redeposition of Cu, may also contribute significantly.

To strengthen this conclusion, we also performed a quantitative analysis of the larger-area FESEM images to assess the extent of surface modification in the presence of different cations. We determined the average size and areal fraction of bright and dark surface regions after CO_2_RR (Figure S4 and Table S1). Notably, the electrodes tested in Li^+^ electrolytes show significantly larger dark regions compared to Cs^+^ and K^+^. Considering that these images were recorded with a secondary electron detector, the darker regions likely correspond to underlying layers exposed by extensive surface dissolution. This quantitative evidence supports our hypothesis that Li^+^ induces deeper dissolution and more pronounced surface restructuring than the other cations under identical CO_2_RR conditions.

Next, ICP analysis was employed to further understand the dependency of Cu dissolution on cations. Typically, Cu dissolution is expected at OCP and higher anodic potentials, where Cu is oxidized to form hydrated Cu^+^/Cu^2+^ ions. Electrolytes were analyzed after subjecting NCs to OCP, −0.8 V, and −1.2 V. Interestingly, ICP results (Figure 3a) show that Cu dissolution in the presence of Cs^+^ is negligible, while it is the highest in Li^+^ under all three conditions. Additionally, it was observed that the concentration of dissolved Cu is the highest at OCP and decreases upon applying a cathodic potential of −0.8 V, potentially due to the redeposition process. Therefore, the application of a cathodic potential likely facilitates the redeposition of dissolved Cu present at OCP onto the electrode surface. However, at −1.2 V dissolution slightly increased, although it remained lower than the values at OCP,^41^ suggesting that there may be another mechanism driving dissolution at this potential.

**Figure 3:**
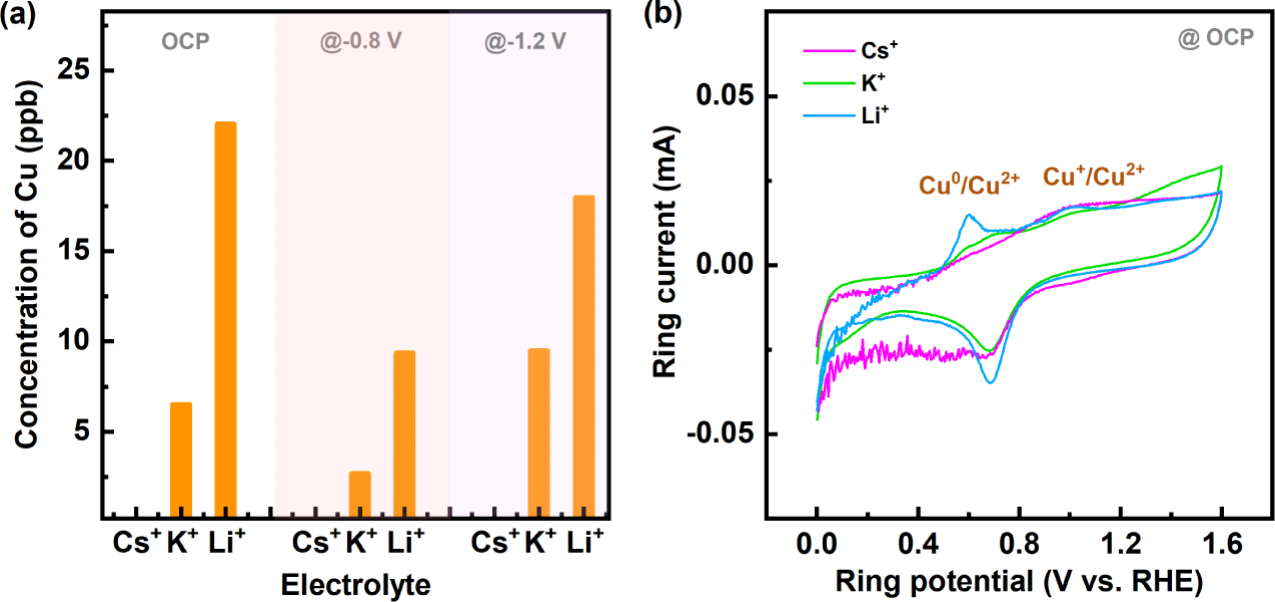
Dissolution of Cu species measured (a) by ex situ ICP method after OCP, and after CO_2_RR at −0.8 and −1.2 V. Cu_2_O NCs were subjected to OCP and cathodic potentials for around 1 min. (b) Oxidation of the dissolved Cu species using RRDE setup (on the Pt ring) in 0.1 M Cs_2_CO_3_, K_2_CO_3_, and Li_2_CO_3_ where disk is at OCP. All indicated potentials are reported vs. RHE.

At OCP condition applied to the disk, species evolving such as CO and OH^–^ are transferred from the disk to the Pt ring due to centrifugal force and detected by the Pt ring through CV scans. The oxidation peak between 0.4 to 0.8 V on the Pt ring is assigned to the oxidation of Cu, which was validated by adding CuCl into the electrolyte by using a Teflon disk (Figure S5). In the presence of CuCl, an initial cathodic current was observed, which is associated with the plating of Cu onto the Pt ring (indicated by the arrow). This was subsequently followed by the oxidation of plated Cu to the Cu^2+^ state.^42–44^ Additionally, Figure S5 illustrates another peak in the CVs of the Pt ring at higher potentials during the anodic scan. This peak, observed between 0.85 and 1.10 V, is attributed to the oxidation of Cu^+^.^45^

RRDE experiment in the electrolytes containing the cations illustrates that the highest amount of dissolved Cu (especially in Cu^2+^ state) is detected on the Pt ring in the presence of Li^+^, followed by K^+^, with a negligible amount in Cs^+^ at OCP (Figure 3b). Overall, the morphological changes through FESEM images of NCs at −1.2 V and findings of ICP and RRDE experiments reveal that Cu species dissolution and redeposition occur most prominently in the presence of Li^+^.

To examine the impact of proton or H_2_ evolution on the dissolution of Cu, we conducted a series of experiments by varying the amounts of Nafion ionomer added to the prepared inks of Cu_2_O NCs. Using the RRDE setup,^46^ CV cycles were recorded at the Pt ring while the drop-casted disk was under OCP conditions (Figure S6). The Cu/Cu^2+^ peak at about 0.60 V demonstrates the dependency of Cu dissolution on the amount of Nafion even at the OCP despite the expectation that Nafion would reduce the dissolution of Cu due to its function as a binder layer, where a higher amount of Nafion is expected to suppress the dissolution of Cu ions.

The other possible expectation at OCP and cathodic potentials would be that more Nafion facilitates the collection and transfer of protons in the vicinity of the electrode surface through its sulfonate $\left({-{\mathrm{SO}}_{3}}^{-}\right)$ groups. This mechanism explains the impact of protons on dissolution, where higher proton concentration leads to the dissolution of Cu species in a greater capacity, with or without cathodic potential. This raises the question of whether the same mechanism is responsible for high dissolution in the presence of Li^+^ and if the dissolution and redeposition of Cu species could explain the observed dependency of CH_4_ and C_2_H_4_ selectivity on cations at high cathodic potentials (−1.2 V).

As discussed earlier, it has been proposed that a higher concentration of protons at lower pH favors CO protonation in the presence of Li^+^, leading to higher selectivity for CH_4_^17^. This assumption was based on the lower current density recorded for Li^+^ containing electrolytes, correlating with lower local pH. However, our results do not support this scenario: at −1.2 V (Figure S1), the current densities for K^+^ and Li^+^ are similar, yet CH_4_ selectivity drops to 0% for K^+^ while C_2_H_4_ reaches its maximum FE of 34%. This clearly indicates that excess protons due to a lower local pH cannot alone explain the observed product trends.

Instead, our results suggest that Li^+^ acts as a proton collector. In other words, its strong Lewis acid nature due to higher charge density,^47^ and rigid, strongly hydration shell enable it to retain more protons near the electrode surface.^48^ The increased proton concentration leads to the surface reconstruction through the dissolution and redeposition of Cu, as confirmed by ICP and RRDE results.

Rather than the local pH effect, the nature of the cation also directly tunes the potential distribution^20^ and local electric field near the Cu_2_O surface.^20^ Weakly hydrated cations such as Cs^+^ and K^+^ can approach closer to the electrode,^15,49–53^ which supports the formation of a stronger interfacial electric field. Cation-induced electrical field stabilizes surface intermediates formed via CO–CO coupling and favors C2 product pathways, as proposed by Malkani and Ringe. Moreover, Li^+^ remaining further from the electrode surface shifts the potential; thus, positively charged protons^54^ can approach the surface more easily, whereas Cs^+^ and K^+^ could repel the protons.

Our EC-SERS results (Figure S7) align with this mechanism: CO remains adsorbed at more negative potentials for Li^+^ but disappears rapidly for Cs^+^ at potentials beyond −0.3 V, indicating faster CO conversion under stronger field conditions^55^ when weakly hydrated cations are present. Moreover, consistent with the findings of Malkani and coworkers^21^ and Resasco and coworkers,^15^ we observe a redshift in the frequency of CO_ad_ as the cation size increases. This redshift induced by weakly hydrated cations (Cs^+^, K^+^) and the associated redistribution of CO adsorption sites arise from changes in the local coordination environment, providing further evidence of surface reconstruction under different cation conditions and stronger interfacial electric fields. This observation is also consistent with the EC-SERS study of An and colleagues,^56^ where surface restructuring generates sites that act as active centers for CO_ad_ and CO–CO coupling,^57^ accompanied by a redshift in the frequency of CO_ad_. Moreover, our observation of stronger CO retention in Li^+^ compared to K^+^ and Cs^+^ is consistent with the recent report by Yang et al.,^58^ which highlights the dynamic migration and stabilization of Cu carbonyl species during CO_2_RR and suggests that mobile surface intermediates may contribute to surface restructuring.

While we measured the double-layer capacitance (Figure S8) and calculated the ECSAs (Table S2) to compare the relative surface area and accessibility, we note that these measurements were conducted in a nonfaradaic region (0.7–0.8 V vs. RHE). Under these conditions, mild surface oxidation or dissolution may occur, which can affect roughness, and the arrangement of cations in the double layer differs significantly from that in the cathodic CO_2_RR regime. Therefore, the measured C_dl_ and calculated ECSA values cannot directly represent the double-layer thickness or local electric field strength and active surface area under actual CO_2_RR operating conditions.

However, the dependency of Cu dissolution on cations may not only arise from alteration of the electrostatic environment but also from accumulation of the cations on the surface. Since dissolution occurs at the same interfacial level, we expect that the amount of Cu dissolved (and subsequent surface restructuring) will be affected by the cation concentration on the surface. The accumulation of weakly hydrated cations (Cs^+^ and K^+^) in the OHP can block the electrode surface^59^ and inhibit the dissolution process. If the electrode surface is covered with cations, Cu is less likely to dissolve through the oxidation process.

While the exact interplay among proton accumulation, double-layer structure, cation blocking, and local field strength is complex, our combined spectroscopic and electrochemical evidence support a coherent picture: the cation effect on CO_2_RR selectivity at high overpotentials cannot be explained by local pH alone but arises from how each cation’s hydration and position modulate the interfacial electric field, local proton availability, and dynamic surface restructuring. These coupled factors ultimately direct the balance between protonation of CO_ad_ and CO–CO coupling, determining whether CH_4_ or C2 products dominate under each electrolyte condition.

To understand how the reconstruction of the Cu surface affects the chemical state of the surface and subsequent product selectivity in the presence of different cations, we investigated the chemical states of the electrode surfaces through ex-situ XAS and XPS. All electrodes were stored in Ar before measurements to minimize the effect of ambient air oxidation.

The Cu L-edge XAS spectra from the fresh Cu_2_O electrode (Figure 4a,b) show a mixture of Cu^+^ and Cu^2+^ states, indicated by features at 931 and 950 eV for Cu^2+^ (Cu­(OH)_2_ or CuO) and at 933.4 and 953.6 eV for Cu^+^ (Cu_2_O)^60^. After CO_2_RR, metallic Cu peaks appear in all electrolytes, but surface hydroxide features are notably suppressed in Li^+^, while they persist more strongly in K^+^ and Cs^+^. This indicates that although metallic Cu redeposits during cathodic polarization in all cases, the retention of hydroxide species depends strongly on cation identity. Cu L-edge XAS results from electrodes after CO_2_RR at −0.8 V indicate similar trends to those at −1.2 V.

**Figure 4:**
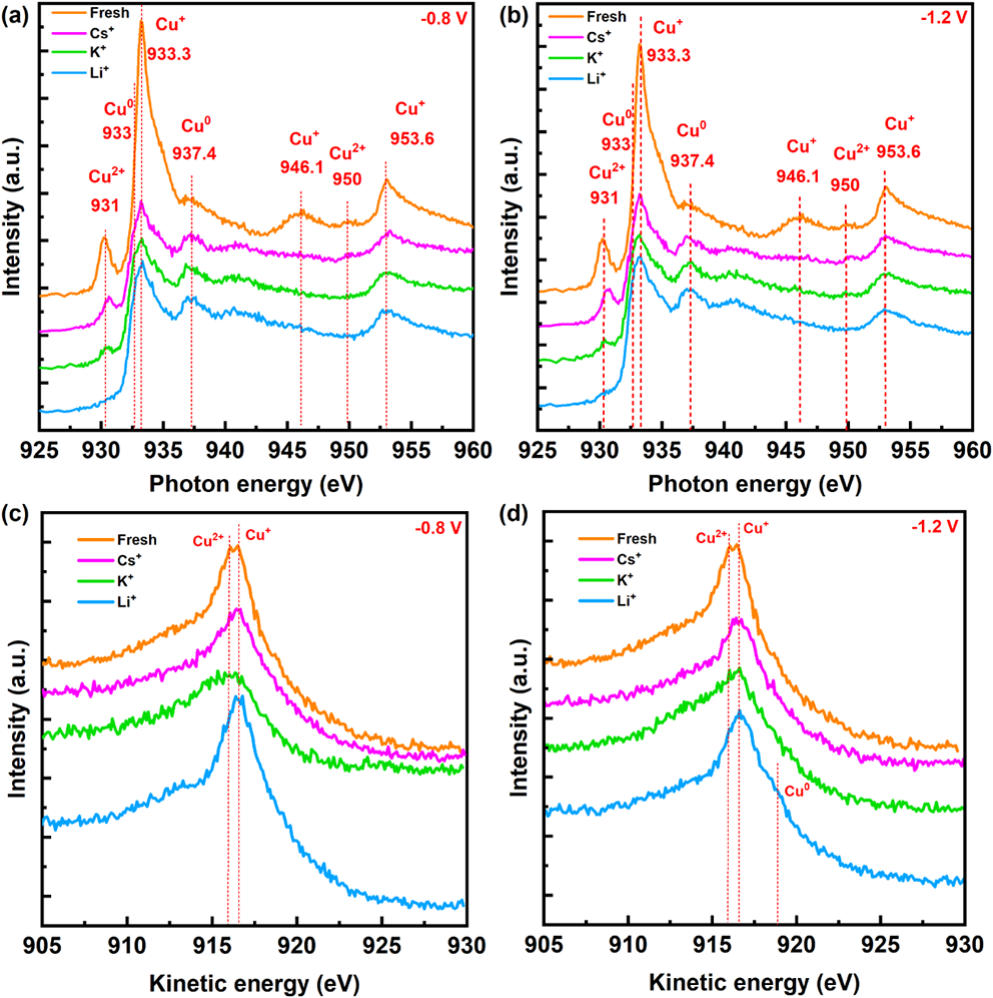
L-edge XAS spectra of Cu_2_O NCs before and after CO_2_RR at (a) −0.8 V and (b) −1.2 V and Cu LMM Auger spectra of Cu_2_O NCs before and after CO_2_RR at (c) −0.8 V and (d) −1.2 V in CO_2_ saturated 0.1 M Cs_2_CO_3_, K_2_CO_3_, and Li_2_CO_3_ electrolytes. All indicated potentials are reported vs. RHE.

Cu LMM Auger spectra (Figure 4c,d) and Cu 2p XPS spectra (Figure S9) revealed that the fresh surface of the electrode contained both Cu^+^ and Cu^2+^ species (Cu­(OH)_2_). Following the application of −0.8 V, electrodes electrolyzed in K^+^ and Cs^+^ still contained Cu^2+^ species, while the electrode tested in the Li^+^ electrolyte mainly consisted of Cu^+^. In this regard, we note that Cu L-edge XAS in fluorescence mode probes a significantly deeper region of the electrode, whereas the Cu LMM Auger peaks from XPS are more surface-sensitive (2–3 nm). Therefore, partial reoxidation of the outermost surface during OCP relaxation or brief air exposure can suppress the Cu^0^ signal in XPS, even when bulk-sensitive XAS still detects metallic Cu beneath the oxide layer. Cu 2p XPS spectra clearly demonstrated the persistent presence of Cu^2+^ on the electrode surface even after CO_2_RR at −1.2 V in the K^+^, compared to other electrodes (Figure S9). Cu LMM Auger peaks identified the presence of Cu^+^ and Cu^0^ states on the surface of electrodes electrolyzed in Cs^+^ and Li^+^. Cu^0^ is distinctive in the presence of Li^+^ at −1.2 V.

XPS data presented in Figure S9 illustrate the stability of the electrode, consisting of mixed oxidation states of Cu^+^ and Cu^2+^, in the presence of Cs^+^ (with higher Cu^+^ content) and K^+^ (with higher Cu^2+^ content). Metallic Cu could not be identified clearly which could be attributed to the fast oxidation of the topmost surface due to air exposure.

EC-SERS was employed to monitor the surface and illustrate the cationic effect on the chemical state of Cu during CO_2_RR at −0.3 V, −0.8 V, and −1.2 V (Figure 5). For comparison purposes, electrodes were examined at dry (no electrolyte) and at OCP (wet) conditions before and at OCP conditions after CO_2_RR.

**Figure 5:**
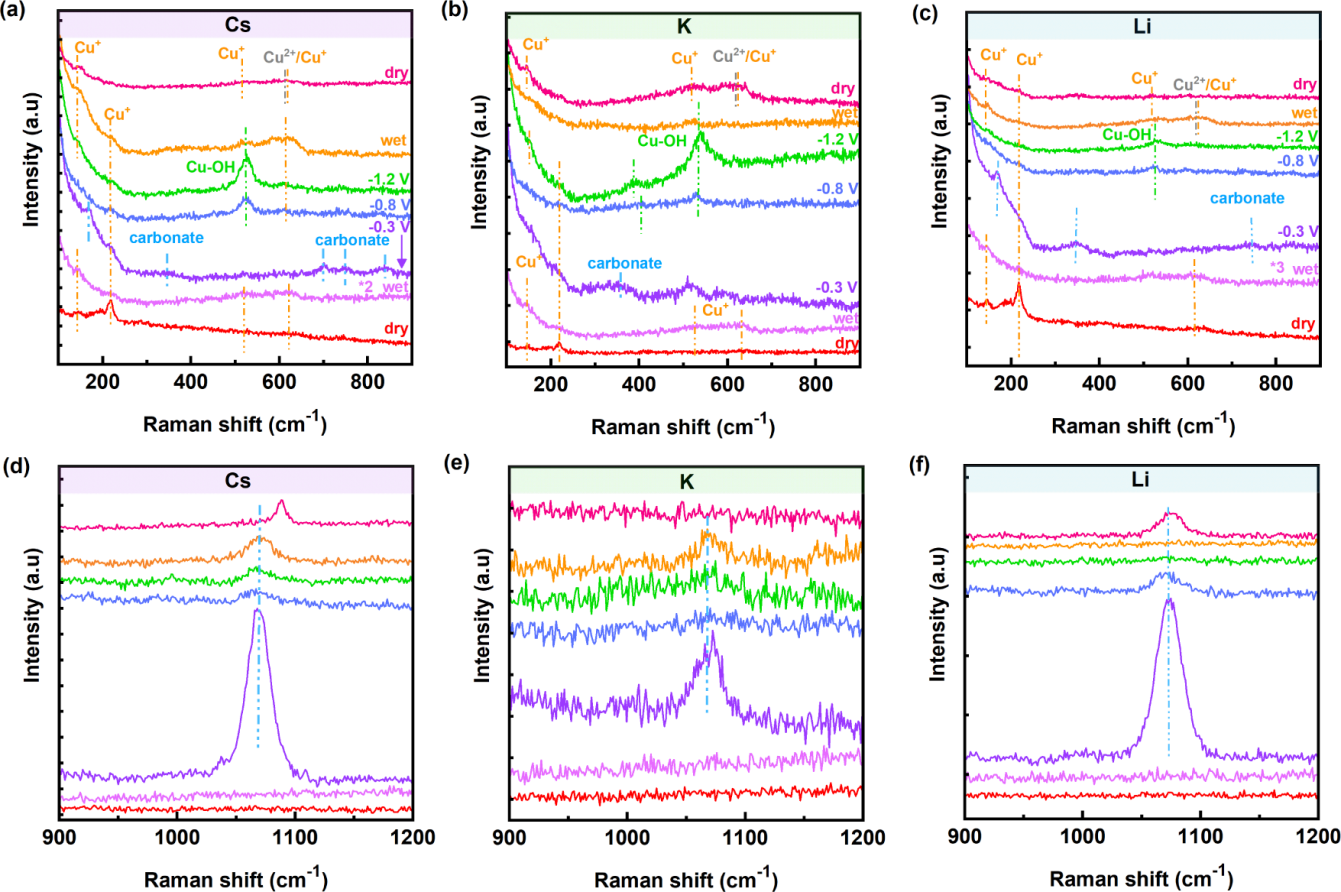
EC-SERS spectra of Cu_2_O NCs before, during, and after CO_2_RR in CO_2_ saturated 0.1 M (a,d) Cs_2_CO_3_, (b,e) K_2_CO_3_, and (c,f) Li_2_CO_3_ electrolytes at low (first row) and high frequency (second row) ranges. All indicated potentials are reported vs. RHE.

These peaks at 145, 220, 412, 520, and 645 cm^–1^ in the Raman spectra represent the Cu^+^ chemical state of the fresh NC electrodes (Figure 5a–c). Details of the vibrational modes are explained in our previous EC-SERS study.^24^ At wet condition, the signal of these peaks is suppressed due to the introduction of electrolytes, while the SERS effect is at its lowest (no Cu oxide reduction). After the application of low cathodic potential (−0.3 V), peaks associated with Cu^+^ were mainly suppressed in Li^+^, while the peak at 220 cm^–1^ stayed relatively as an intense shoulder in the Cs^+^ electrolyte and less intense in K^+^ electrolyte. Other features observed at this potential are carbonate-related species. Peaks at 350 and 700 cm^–1^ are associated with carboxylate,^61,62^ and peaks at 750, and 835 cm^–1^ are assigned to carbonate $\left({\mathrm{CO}}_{3}^{2-}\right)$ species, which are clearly detected in all spectra taken at −0.3 V. EC-SERS spectra measured at −0.3 V in higher frequency regions (Figure 5d–f) include monodentate ${\mathrm{CO}}_{3}^{2-}$ with the highest intensity for Cs^+^ and Li^+^ electrolytes, and lowest for K^+^.^62^ This peak generally arises while the Cu oxide phase partially or fully reduces. Therefore, we can conclude here that in the presence of K^+^, Cu oxides/hydroxides have covered the surface, in agreement with the XPS findings.

At −0.8 V, the shoulder peak at 220 cm^–1^ assigned to Cu_2_O, despite partially suppressed, was observed for all cations. Additionally, another peak at 614–620 cm^–1^ was detected, which is assigned to both CuO and Cu_2_O species.^24^ The peak at 530 cm^–1^, which has emerged specifically at higher cathodic potentials, is representative of the Cu–OH band in the presence of all three cations.^61,63,64^ The Cu–OH feature enhances at the next higher cathodic potential (−1.2 V) likely due to the higher reduction reaction rates which cause higher OH^–^ concentration. At −1.2 V, the intensity of the Cu–OH bands clearly varies among different cations, however, only one intensity cannot solely be used to interpret the abundance of the species. Therefore, the intensity ratios of the Cu–OH to ${\mathrm{CO}}_{3}^{2-}$ peaks were calculated and plotted in Figure 6. This ratio shows the dependency of the Cu–OH peaks on the type of the cation. Interestingly, this ratio follows the dependency of FE of C_2_H_4_ on the cations, highlighting the enhanced efficiency of that in the presence of K^+^, then Cs^+^. The gap between Cs^+^ and Li^+^ can be justified by the presence of Cu oxide species, more abundant for Cs^+^ electrolyte and metallic Cu for Li^+^, which aligns well with XAS and XPS findings.

**Figure 6:**
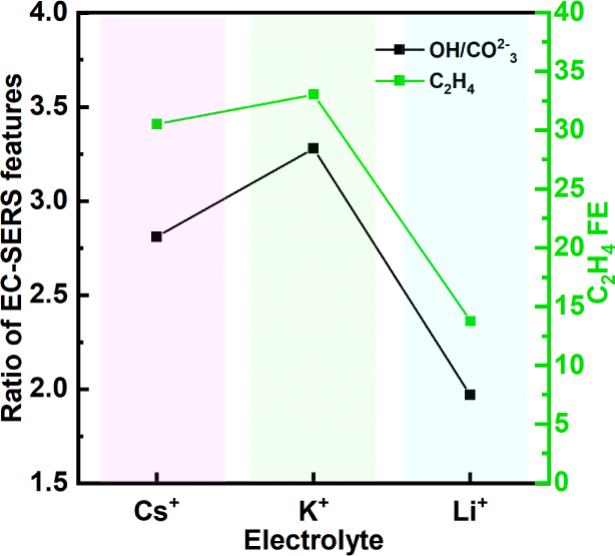
Relation between relative abundance (peak area ratio) of ${\mathrm{OH/CO}}_{3}^{2-}$ and C_2_H_4_ FE as a function of cation, both measured and calculated at −1.2 V vs. RHE.

The positive effect of OH^–^ bonding to Cu atoms on CO_2_RR to C2+ products, through a decrease in the activation energy barrier for the CO dimerization step, has been reported by the Sargent group.^32^ However, in our study, XPS and XAS results showed that an abundance of OH^–^ species is also concomitant with the formation of a Cu­(OH)_2_ layer. Another study of Cu electrodes in a pulsed CO_2_RR^65^ system demonstrated that a higher concentration of adsorbed OH^–^, where protons are repelled during the anodic potential, increases the selectivity for C2+ products. This selectivity in the presence of adsorbed OH^–^ is attributed to the stabilization of CO and easier dimerization.

The existence of surface oxide and hydroxide species at varied potentials during CO_2_RR depicted by EC-SERS spectra suggests that Cu_2_O NCs are not fully metallic. EC-SERS spectra acquired at OCP after CO_2_RR show the formation of Cu^+^ consuming the OH^–^ species on the surface of the Cu^63^ through the following reactions:^31,66^

$${\mathrm{Cu}}^{+}+{\mathrm{OH}}^{-}\to \mathrm{Cu}{\left(\mathrm{OH}\right)}_{\mathrm{ad}}$$

$$2\mathrm{Cu}{\left(\mathrm{OH}\right)}_{\mathrm{ad}}\to {\mathrm{Cu}}_{2}O+H_{2}O$$

On the basis of our ICP, RRDE, XAS, XPS, EC-SERS, and microscopy results, we conclude that CO_2_RR selectivity on Cu_2_O NC electrodes is strongly governed by the chemical state and structural evolution of the surface, which are in turn modulated by the cations present. Our findings show that the interfacial electric field, shaped by different cations, alters proton distribution and thus drives Cu dissolution, reconstruction, and chemical state changes. Specifically, Li^+^ allows more protons to accumulate at the surface, promoting dissolution and redeposition of metallic Cu, which favors CH_4_ formation. In contrast, Cs^+^ and K^+^ block the surface more effectively, stabilizing Cu­(OH)_2_ layers that correlate with higher C_2_H_4_ faradaic efficiency. This trend aligns with previous studies showing that Cu­(OH)_2_ phases enhance C2 product formation during CO_2_RR.^8,32,60,67–69^ This oxidation state trend, supported by XAS, XPS, and EC-SERS, emphasizes the key role of hydroxides in promoting C–C coupling. Combining ICP, RRDE, FESEM, EC-Raman, and GC results, we now present a complete mechanistic picture: cations, by tuning the interfacial field and surface structure, determine the distribution of adsorbed intermediates and thus the balance between CO protonation and CO–CO coupling pathways. Together, this multistep evidence clarifies how cation identity precisely directs CO_2_RR product selectivity.

## Conclusion

This study explores the intricate relationship between cation types, Cu surface states formed over the dissolution and restructuring, and product selectivity during CO_2_RR over Cu oxide nanocubes. The research demonstrates that cations significantly influence both the dissolution and redeposition of Cu species, which in turn dictates the chemical state of the electrode surface and eventually the selectivity of CO_2_RR products. At higher cathodic potentials, particularly in the presence of Li^+^, the Cu surface experiences pronounced dissolution. This is attributed to the higher proton concentration near the electrode surface, which is facilitated by enhanced local proton activity caused by Li^+^. Li^+^, with a rigid hydration shell, tends to stay further from the electrode, allowing protons to accumulate near the surface. This accumulation enhances Cu dissolution. The dissolution process involves the formation of Cu ions (Cu^+^ and Cu^2+^), which are then redeposited as metallic Cu under the applied cathodic potential, leading to significant restructuring of the Cu nanocubes. Conversely, in the presence of Cs^+^ and K^+^, the dissolution of Cu is markedly lower. These weaker hydrated cations, reside closer to the electrode surface, creating a shielding effect that inhibits the dissolution of Cu. This is evident in the minimal Cu dissolution observed in these electrolytes, as confirmed by ICP and RRDE measurements. The reduced dissolution in the presence of Cs^+^ and K^+^ results in a more stable surface, where mixed valence states of Cu (Cu^+^ and Cu^2+^) persist.

This restructuring, driven by the interaction of cations with the electrode surface, influences the chemical state of the Cu nanocubes and the selectivity of the CO_2_RR products. XAS, XPS, and EC-SERS results indicate a higher intensity of Cu­(OH)_2_ in the presence of K^+^ and Cs^+^, correlating with a higher FE for C_2_H_4_. In contrast, a metallic Cu surface, observed in the presence of Li^+^, is more active for C1 products. These results highlight the significance of choosing the appropriate cations to adjust the surface chemistry of Cu/Cu oxide electrodes, which in turn influences the product selectivity of CO_2_RR. Understanding this relationship offers critical insights for optimizing electrocatalysts and electrolyte properties to achieve the desired CO_2_RR products.

## Supplementary Material



## Notes

Comparison of current densities, morphological characterization of Cu_2_O NCs before and after CO_2_RR, RRDE reference measurements, detection of dissolved Cu species by RRDE, EC-SERS spectra of Cu_2_O NCs, ECSA measurements, and Cu 2p XPS of Cu nanocube electrodes before and after CO_2_RR (PDF)
