# Supplementary material for: Cations Responsible for Varied CO2RR Product Selectivity at High Overpotentials over Cu2O Nanocubes
Source: ACS Appl Mater Interfaces. 2025 Sep 2;17(36):50676–85. doi: 10.1021/acsami.5c09803 (PMC12442015; doi:10.1021/acsami.5c09803)
Supplement: Supplementary file 1 [file am5c09803_si_001.pdf]

Supporting Information

Cations Responsible for Varied CO_2RR Product Selectivity at High Overpotentials over Cu_2O Nanocubes

Saeede Tafazoli[1,2], Azra Şekercioğlu[2,3], Hamaneh Zarenezhad[2], Başak Ataş[2,3], Amin Mohammadpour[2,3], Timuçin Balkan[3], Sarp Kaya[2,3,*]

[1] Materials Science and Engineering, Koç University, 34450 Istanbul, Türkiye

[2] Koç University Hydrogen Technologies Center (KUHyTech), 34450 Istanbul, Türkiye

[3] Department of Chemistry, Koç University, 34450 Istanbul, Türkiye

*Corresponding author: sarpkaya@ku.edu.tr (Sarp Kaya)

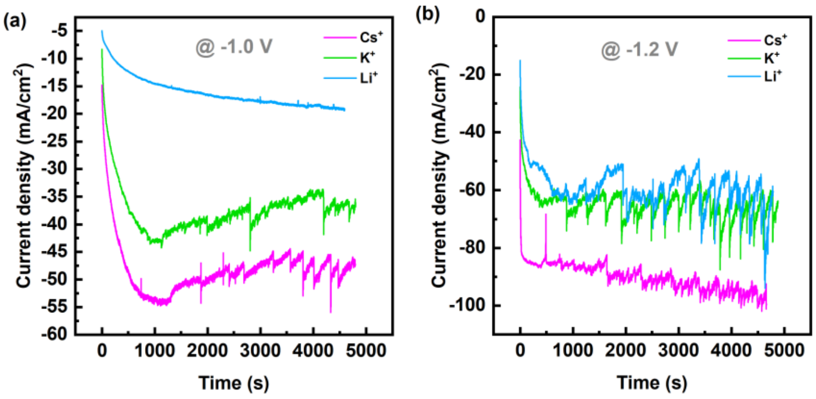

Figure S1. Comparison of current densities recorded during CO_2RR in CO_2 purged 0.1 M Cs_2CO_3, K_2CO_3, and Li_2CO_3 electrolytes at (a) -1.0 V, and (b) -1.2 V. All indicated potentials are reported vs. RHE.

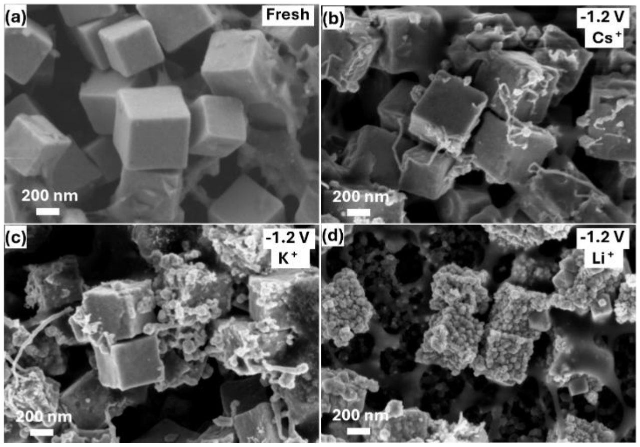

Figure S2. Morphological characterization of 400 nm Cu_2O NCs, (a) before, and after CO_2RR in CO_2-purged 0.1 M (b) Cs_2CO_3, (c) K_2CO_3, and (d) Li_2CO_3 electrolytes at -1.2 V.

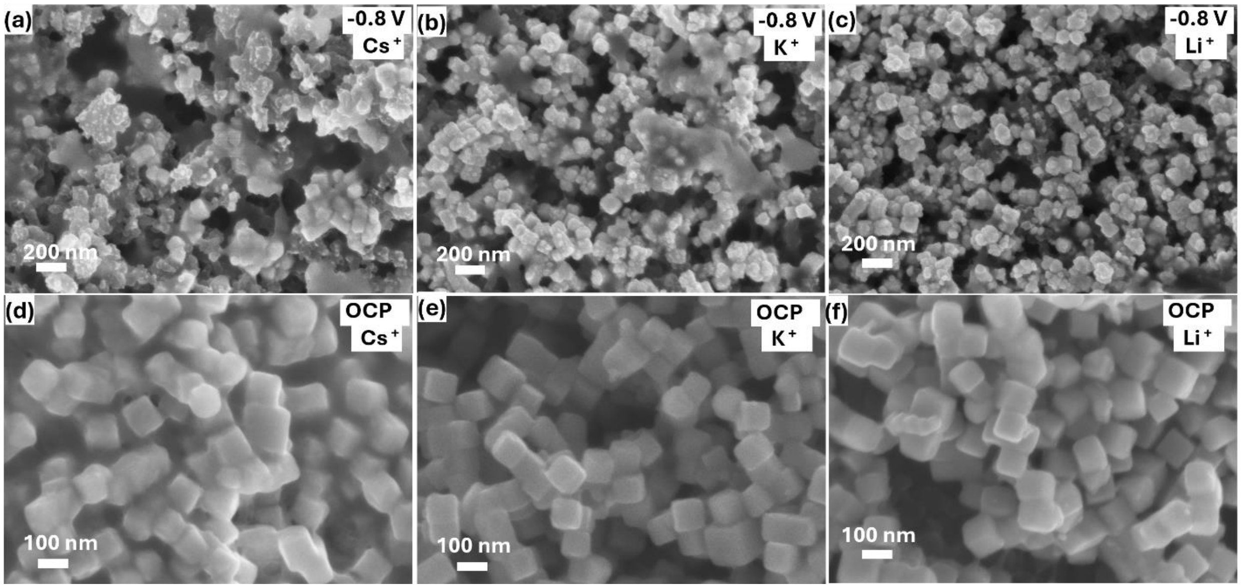

Figure S3. Morphological characterization of Cu_2O nanocubes after CO_2RR at -0.8 V and before CO_2RR at OCP condition in CO_2-purged 0.1 M electrolytes of (a) and (d) Cs_2CO_3, (b) and (e) K_2CO_3, and (c) and (f) Li_2CO_3. All indicated potentials are reported vs. RHE.

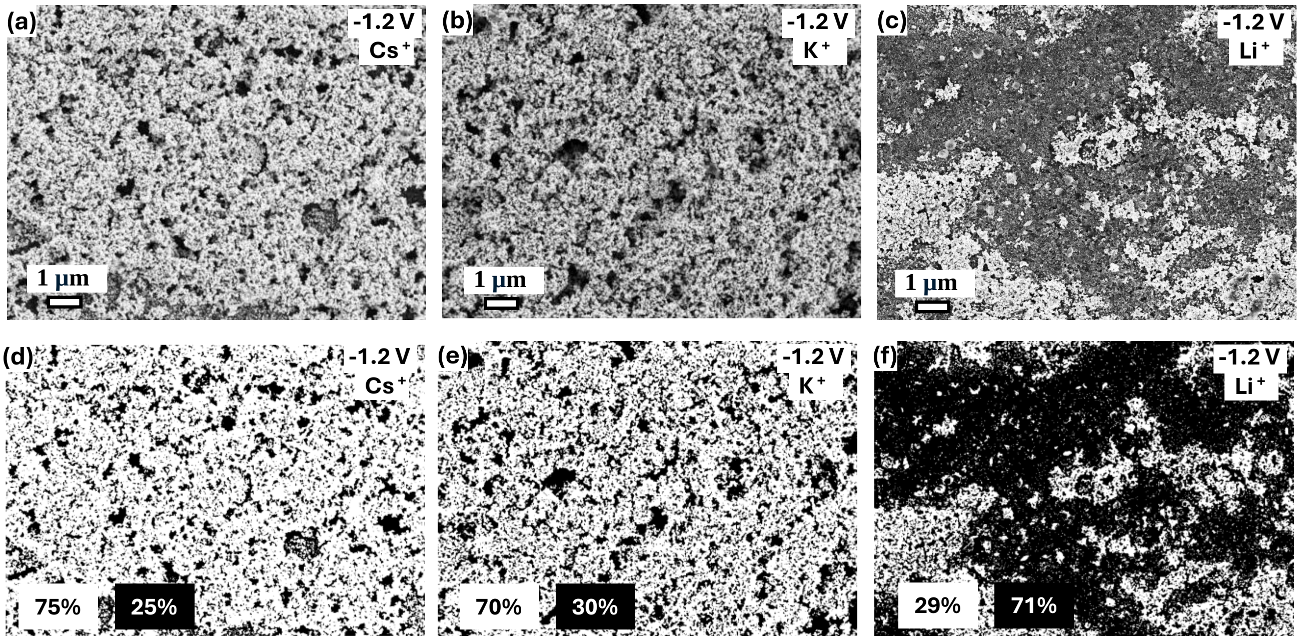

Figure S4. Morphological characterization of Cu_2O nanocubes after CO_2RR at -1.2 V (vs. RHE) in CO_2-purged 0.1 M electrolytes of (a) and (d) Cs_2CO_3, (b) and (e) K_2CO_3, and (c) and (f) Li_2CO_3. The large area SEM images from the surface of the electrodes (upper row) have been analyzed using ImageJ software (lower row). Areas are quantified by integrating black and white distributions in histogram plots.

Table S1. Percentage of bright and dark areas detected by the ImageJ software from the SEM images corresponding to the measurement in Cs_2CO_3, K_2CO_3, and Li_2CO_3.

	Bright area (%)	Dark area (%)
Cs^+	75	25
K^+	70	30
Li^+	29	71

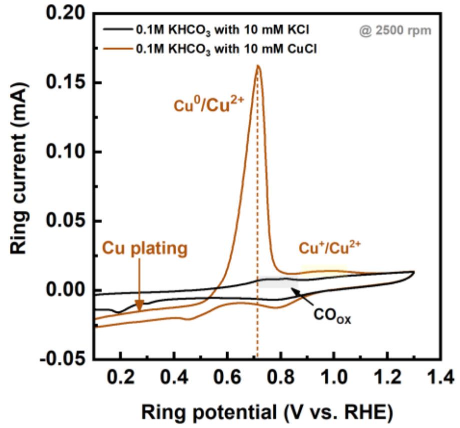

Figure S5. CVs recorded at the Pt ring in the presence and absence of 10 mM CuCl and 10 mM KCl in CO_2-purged 0.1 M $KHCO_3$, with the RRDE tip containing the Pt ring and Teflon disk.

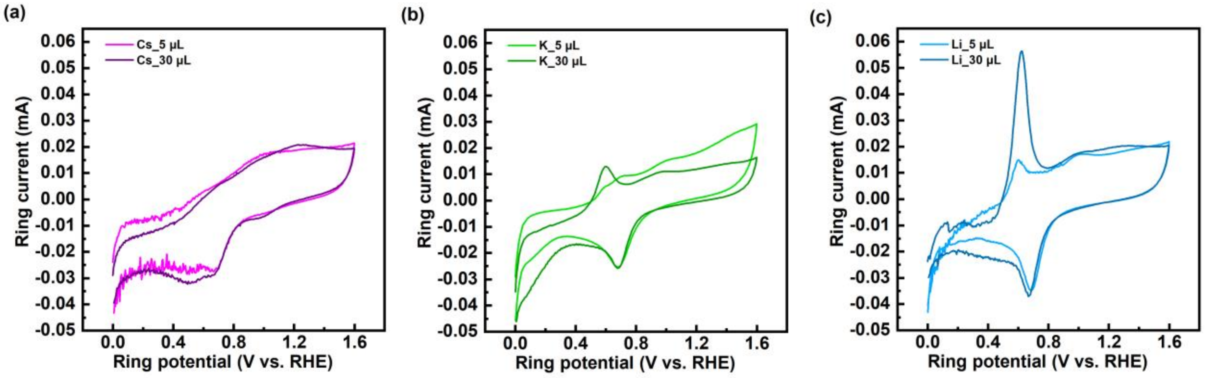

Figure S6. Detection of dissolved Cu species into CO_2 purged 0.1 M (a) Cs_2CO_3, (b) K_2CO_3, and (c) Li_2CO_3 electrolytes by the Pt ring of the RRDE setup. The rotation rate was 1600 rpm and the potential scan rate of the Pt ring was 50 mV/s and disk was at OCP. Cu_2O NC inks for electrodes were prepared using different amounts of Nafion (5 and 30 μL). The constant collection efficiency at different rotation rates was considered.

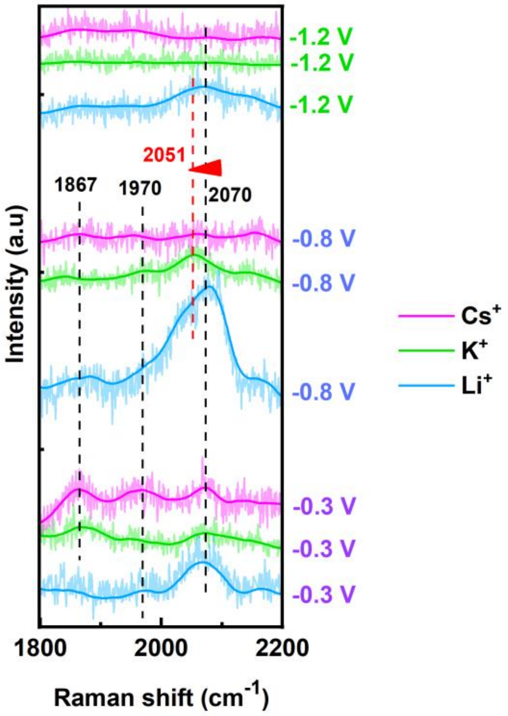

Figure S7. EC-SERS spectra of Cu_2O NCs at (a) -0.3 V, (b), -0.8 V, and (c) -1.2 V during CO_2RR in CO_2 saturated 0.1 M Cs_2CO_3, K_2CO_3, and Li_2CO_3 electrolytes at high frequency (CO adsorption) ranges. All indicated potentials are reported vs. RHE.

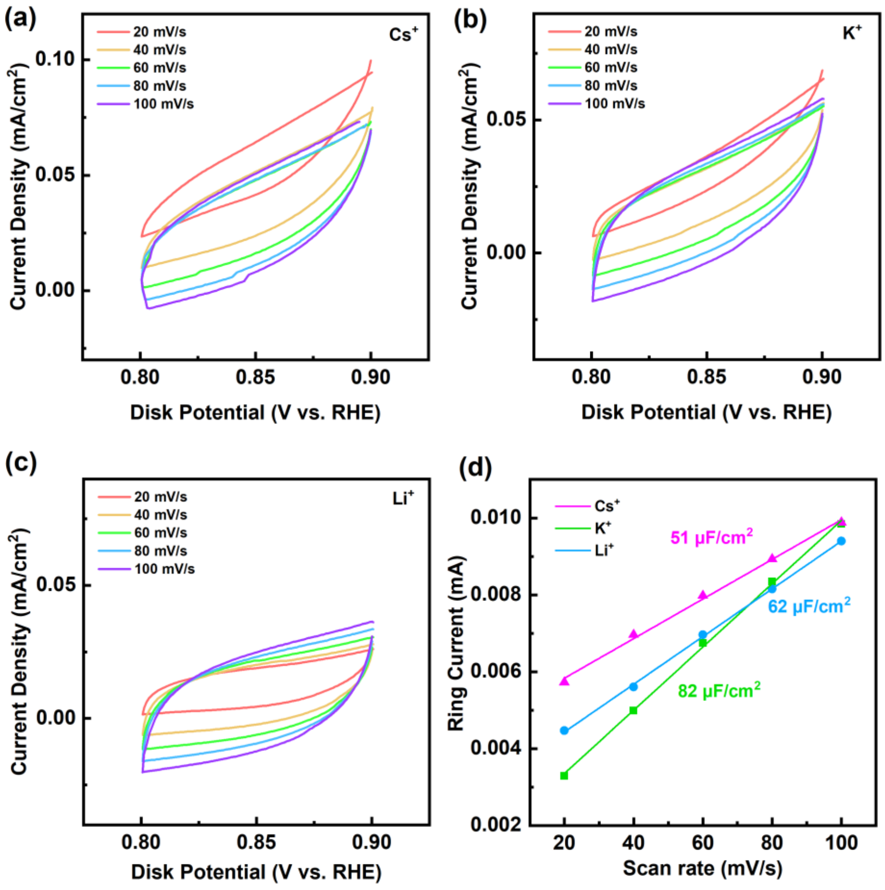

Figure S8. CVs of as-prepared NCs in CO_2-purged 0.1 M electrolytes of (a) Cs_2CO_3, (b) K_2CO_3, and (c) Li_2CO_3. (d) C_{dl} derived from CV measurements of all three electrodes in different electrolytes.

Table S2. ECSA values estimated from CV cycles at different scan rates of as-prepared NCs in CO_2-purged 0.1 M electrolytes of (a) Cs_2CO_3, (b) K_2CO_3, and (c) Li_2CO_3.

	Cs^+	K^+	Li^+
ECSA	1.27	2.05	1.55

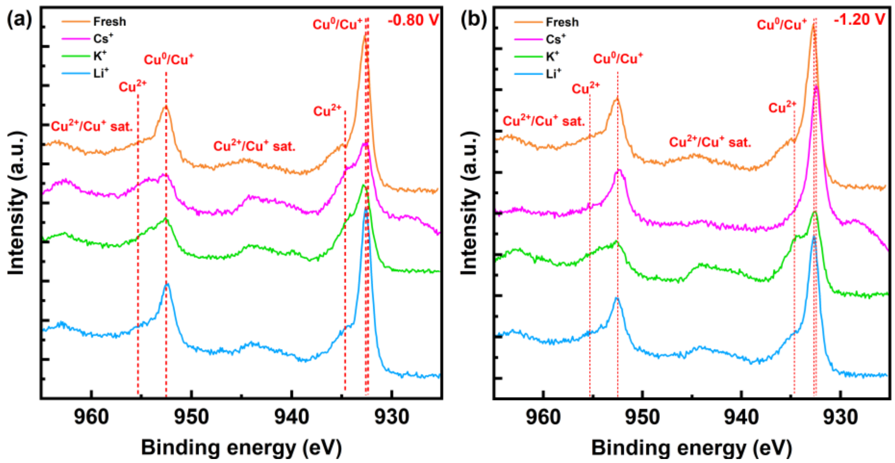

Figure S9. Cu 2p XPS of Cu nanocubes electrodes before CO_2RR, and after CO_2RR in CO_2-purged 0.1 M electrolytes of Cs_2CO_3, K_2CO_3, and Li_2CO_3 at OCP, (a) -0.8 V, and (b) -1.2 V. All indicated potentials are reported vs. RHE.
